# Supplementary material for: Unique morphogenetic signatures define mammalian neck muscles and associated connective tissues
Source: eLife. 2018 Nov 19;7:e40179. doi: 10.7554/eLife.40179 (PMC6310459; doi:10.7554/eLife.40179)

Information on the use of the interactive 3D PDF document

Color code of skeletomuscular structures

- Skeletal components
- Muscles of cranial mesoderm origin
- Muscles of anterior somitic origin
- Muscles of posterior somitic origin

Navigation

Putting the mouse cursor over the interactive window, a menu with additional options will appear

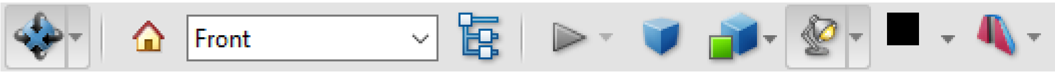

Selection of interaction with the model (rotate , turn , pan , zoom etc.)
Possibility of 3D measurement tool

Front — List of defined views for the current 3D model

Change of pre-defined lights

Change of pre-defined background color

Change of pre-defined rendering

Virtual cross section on the model

Selection of preset views

- Click on the color button to see the corresponding structure.
- Click on the preset views to see the corresponding perspective.
- Click on button to show/hide the model tree on the left part of the document. In the model tree, you can show/hide the different structures of the 3D model.

Example of a model tree:

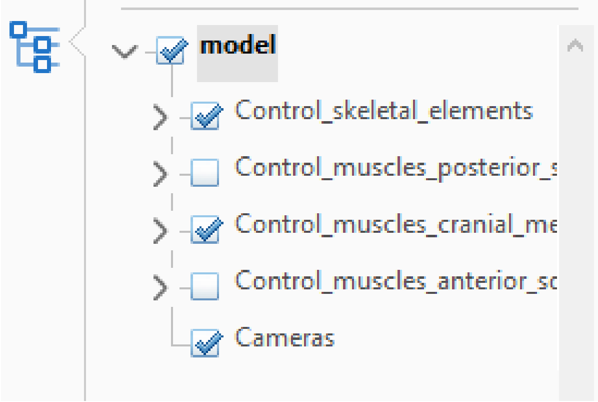

Corresponding 3D model:

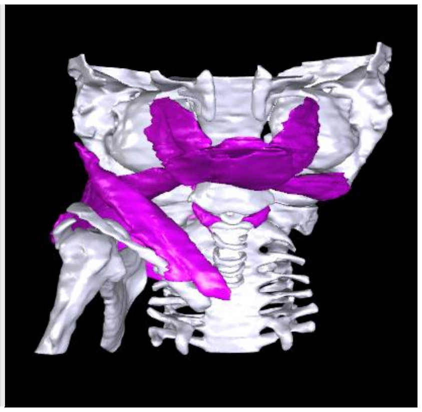

www.3Dpdfmaker.com

Technical note

To view interactive PDF files, you need to use the free standard Adobe Reader®/Acrobat Reader DC (www.adobe.com/downloads/). If you see a warning message in the yellow dialog box on the file opening, you can activate the content by pressing the button "Options", otherwise you can enable 3D data for all documents in the preferences dialog box (Edit / Preferences / 3D & Multimedia / check the box "Enable playing of 3D content".

Annotated static views

Lateral views | **Frontal views** | **Medio-lateral views**

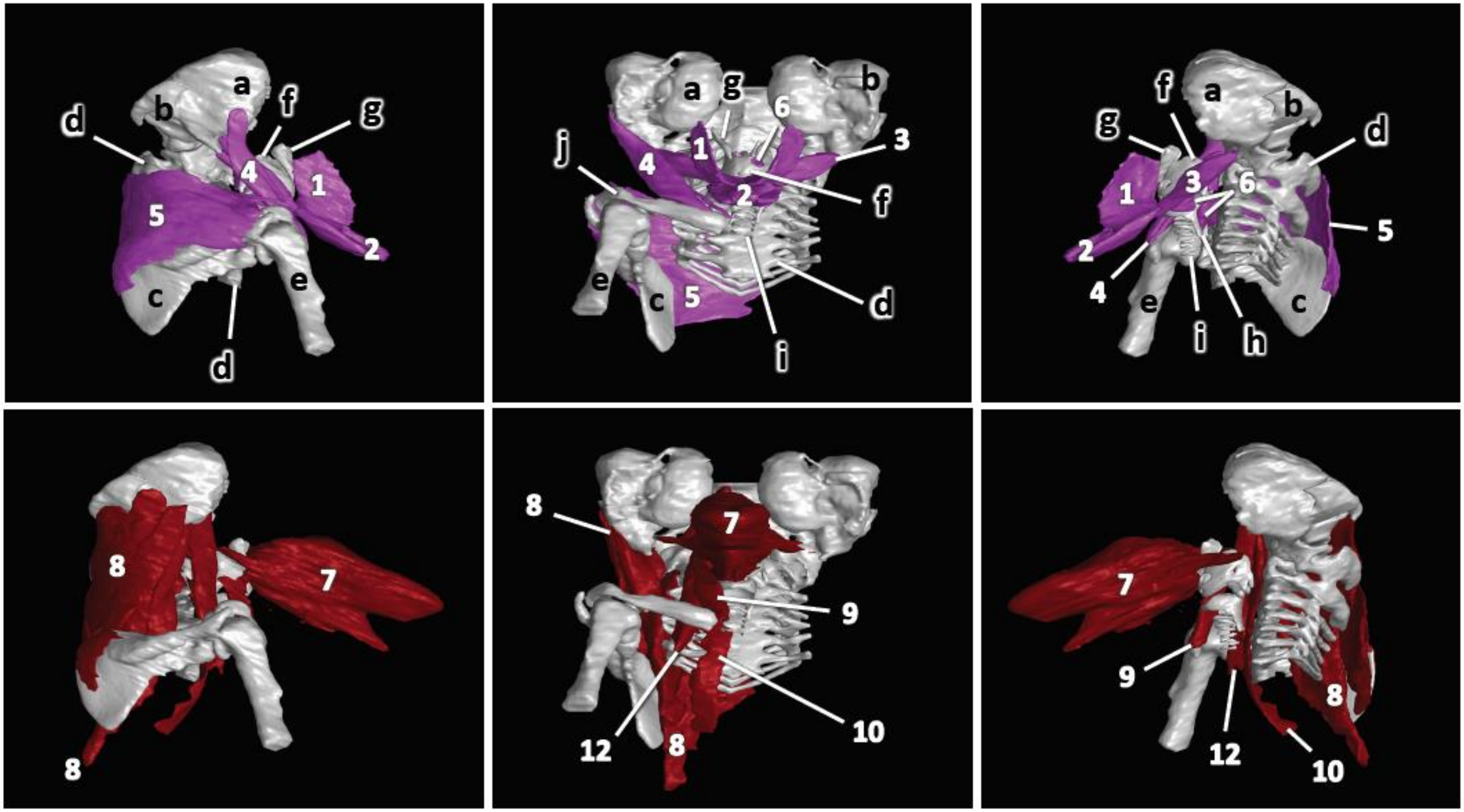

Skeletal components | Cranial mesoderm muscles | Anterior somitic muscles | Posterior somitic muscles

Skeletal components

a. otic capsule
b. skull base*
c. scapula*
d. cervical vertebrae*
e. humerus*
f. thyroid cartilage*
g. hyoid bone*
h. cricoid cartilage
i. tracheal rings
j. clavicle*

Muscular components

1. mylohyoid
2. anterior digastric
3. posterior digastric
4. sternocleidomastoid*
5. acromiotrapezius
6. laryngeal muscles*
7. tongue muscles**
8. epaxial musculature*
9. infrahyoid muscles*
10. longus colli
11. scapular muscles*
12. longus capitis

* intrinsic and extrinsic tongue muscles

* affected in *Pax3* mutants

* missing in *Pax3* mutants

Interactive 3D neck reconstruction of a *Pax3*-inactivated foetus (E18.5)

Structures

All structures

Skeletal components

Muscles of cranial mesoderm origin

Muscles of anterior somitic origin

Muscles of posterior somitic origin

Preset views

ventral

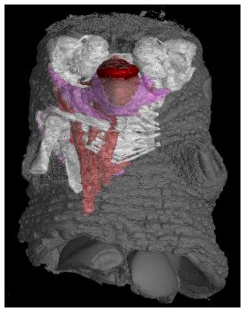

dorsal

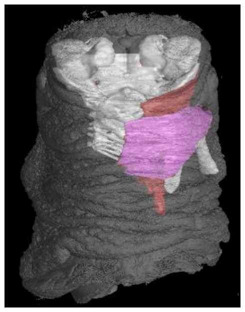

lateral

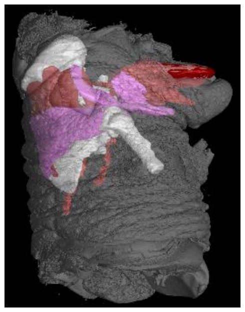

medial

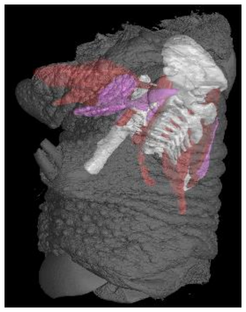

rostral

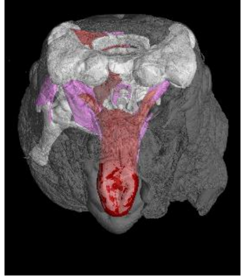

caudal

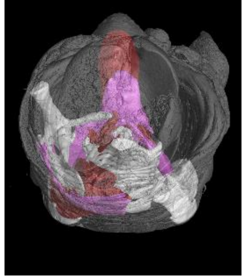

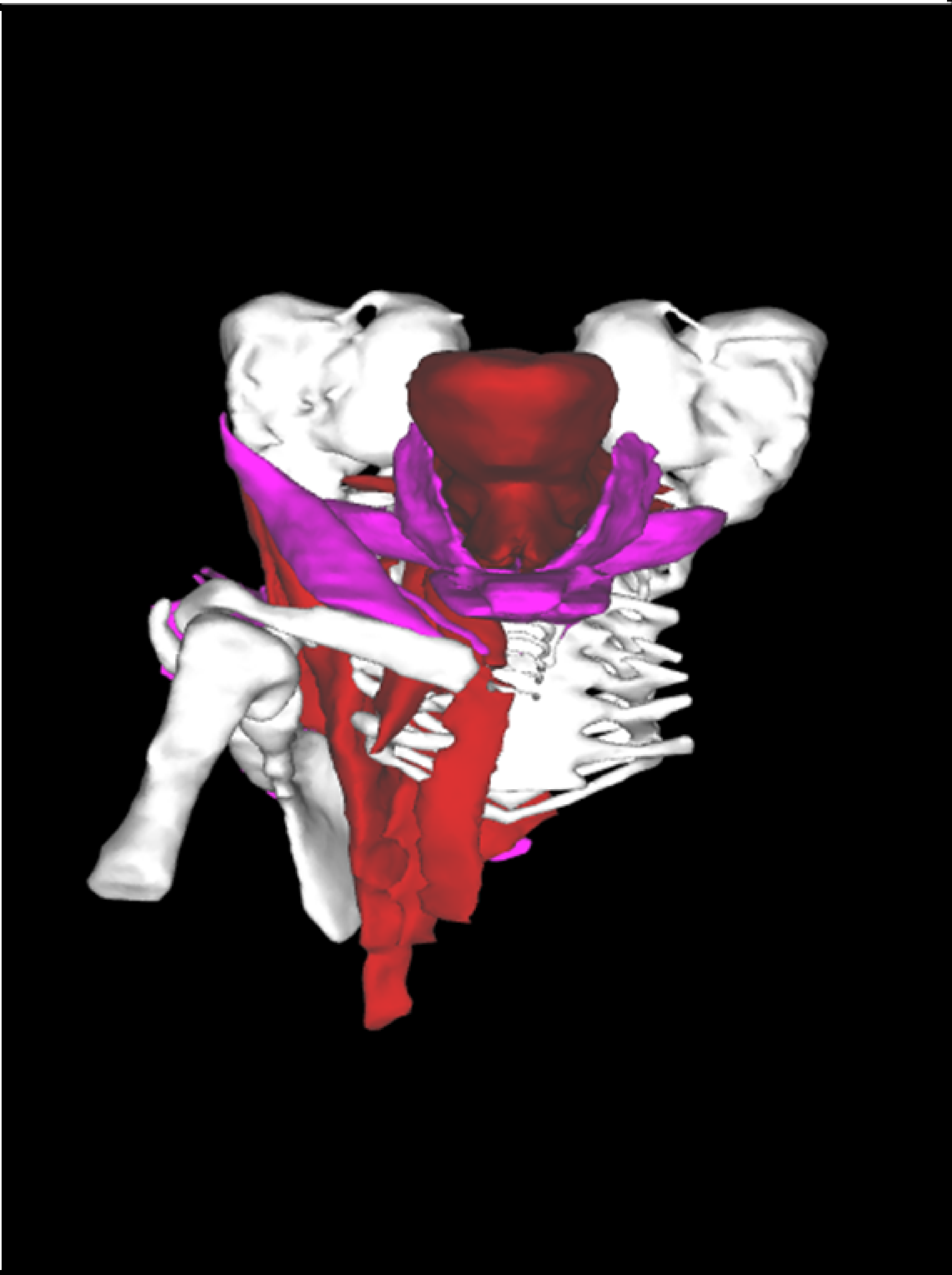

Supplement: Supplementary file 3. — Download PDF for full details. [file elife-40179-supp3.pdf]
